# Co-occurring intimate partner violence, mental health, and substance use problems: a scoping review

**DOI:** 10.3402/gha.v7.24815

**Published:** 2014-11-20

**Authors:** Robin Mason, Susan E. O'Rinn

**Affiliations:** 1 Women's College Hospital, Women's College Research Institute, Toronto, Ontario, Canada; 2 Dalla Lana School of Public Health and Department of Psychiatry, University of Toronto, Toronto, Ontario, Canada

**Keywords:** intimate partner violence, mental health, substance use, education, scoping review

## Abstract

**Background:**

Intimate partner violence (IPV) is a pervasive, serious problem detrimental to the health of untold numbers of women. In addition to physical injuries that may be sustained, IPV has been significantly associated with mental health challenges including substance use problems. The problems are complex, highly correlated with each other, and bidirectional in nature. Although as many as 50% of women in mental health and between 25% and 50% of women in substance abuse treatment programs report IPV, frontline workers in all three sectors state they lack the training to address these co-occurring problems.

**Objective:**

To determine what frontline IPV, mental health, and substance use workers need to know in order to provide appropriate care to women experiencing co-occurring IPV, mental health and/or substance use problems.

**Design:**

Using Scholars Portal OVID, Medline and OVID PsycINFO and combinations of significant terms, we conducted a scoping review of articles published between 2005 and 2014.

**Results:**

An initial 4017 records were retrieved (3484 from Scholars Portal, 272 from Medline, 261 from PsycINFO). After applying inclusion and exclusion criteria, 35 articles were reviewed. Of these, 14 examined the relationships among IPV, mental health, and substance use; 7 focused on IPV and mental health; 14 looked at IPV and substance use.

**Conclusions:**

Although education and training frequently figured among the recommendations in the reviewed articles, specific content for proposed education or training was lacking. The most frequently occurring recommendations focused on the need to develop better collaboration, coordination, and integration across IPV, mental health and addiction treatment services.

Intimate partner violence (IPV) is a common, serious problem with deleterious impacts on the health of women. In 2002, the World Health Organization deemed it a public health epidemic (1) and, recently, under the Affordable Care Act (2), the United States introduced insurance to cover screening and brief counseling costs for IPV in routine healthcare practices.

Although IPV may be perpetrated by either men or women, the majority of victims are women (3) and women report experiencing more serious forms of violence and more serious consequences of violence than do men (4, 5), including an increased risk of developing mental health and substance use problems (6).

IPV occurs at alarming rates worldwide. For example, in the United States, an estimated 35.6% of women experience rape, physical violence, and/or stalking in their lifetime within the context of an intimate relationship (7), whereas in Canada more than 25% experience IPV at some point in their lives (8). However, when looking at prevalence rates among ‘treatment’ populations, the numbers of victims/survivors are even more daunting. A recent review article noted that ‘on average, over half of women seen in a range of mental health settings either currently are, or have been abused by an intimate partner’, although rates vary widely across studies (9).

The relationship between IPV and mental health problems has been well-documented with depression, dysthymia, suicidality, generalized anxiety disorder, phobias, and posttraumatic stress disorder (PTSD) all associated with the experience of IPV (10–15). Some studies reported worse outcomes associated with greater frequency and severity of IPV (16), whereas others reported that even so-called low levels of violence (such as pushing or shoving) are associated with depression (17, 18). Psychological or emotional abuse has been associated with depression, low self-esteem, and PTSD (19, 20). In at least one study, more than 50% of the women who experienced IPV suffered some mental health problem and nearly 75% of the women who experienced ‘severe’ IPV had one or more diagnosed mental health disorders (21), leading one researcher to note that ‘among treatment-seeking women diagnosed with a severe mental disorder, there is a ‘high risk’ of IPV’ (22).

Among American women in substance abuse treatment programs, reported prevalence rates for exposure to IPV have ranged from 20% to 57% (23–26). There are scant Canadian data but results from a sample of women drawn from nine substance abuse treatment centers in Ontario, Canada, reported 56% had experienced adult physical abuse (27). One other substance abuse treatment program reported that 65% of women in the program had experienced some form of violence in their adult years (although not necessarily IPV) (28).

The relationships among IPV and substance use are complex with some studies reporting increased substance use following IPV (29–31), whereas others report that substance use may lead to higher rates of IPV (29, 32). Recent studies suggest that both propositions are true: IPV is associated with increased substance use and substance use is associated with an increased risk of IPV. Thus, the relationships are both complex and bidirectional; just as with mental health problems, substance use may be a response to the effects of abuse (29, 32) and/or make women more vulnerable to abuse (29–31).

Despite the clear relationships among IPV, mental health, and substance use problems, programs and practices have evolved along discrete lines governed by different paradigms (33), professions, languages (33, 34), training models (35), and funding streams (social or justice services versus healthcare). Yet, the frequent co-occurrence of IPV with mental health and/or substance use problems suggests that clinicians and frontline providers who work with women in IPV, mental health, or addiction treatment settings should be trained to recognize and appropriately respond to women who experience these co-occurring problems. Early in 2010, we completed a review of the literature (2005–2010) to guide the development of an evidence-informed curriculum relevant to frontline providers who work in IPV, mental health, or addiction treatment settings. The review was updated in 2014 (2010–2014) and the integrated results from 2005 to 2014 are presented here.

## Objective

Our objective was to answer the question ‘what do clinicians and frontline workers need to know in order to provide appropriate care to women who may experience co-occurring IPV, mental health and/or substance use problems?’

## Method

An initial scoping review of articles published between 2005 and 2010 was completed in January 2010 using Scholars Portal databases.^1^ This was updated in August 2014 to include articles published between 2010 and 2014 using OVID Medline and OVID PsycINFO databases. The Scholars Portal databases search used combinations of the following terms: ‘intimate partner violence’, ‘domestic violence’, ‘intimate partner abuse’, ‘spousal abuse’, ‘marital violence’, ‘violence against women’, ‘mental health’, ‘addiction’, ‘substance abuse’, plus any of the following: ‘treatment’, ‘treatment modalities’, ‘trauma informed’, ‘education’, ‘curriculum’, and ‘training’. The search of OVID Medline and PsycINFO databases used similar terms (see Appendix 1 for the literature search strategy).

Included were English language articles focused on: women; IPV; mental health and/or substance use; and treatment/education/training (see above search terms). No geographic limitations were applied.

Excluded were articles that failed to address IPV and one of the co-occurring (mental health or substance use) problems and treatment/education/training issues. Also excluded were studies focused on male victims, perpetrators, children, adolescents, couples and men's use of drugs/alcohol (except when related to women's experiences of IPV). Duplicates as well as book titles were also eliminated.

A scoping review is a rapid, systematic examination of the literature on a topic area. Scoping reviews aim to rapidly identify the key concepts within a specific research domain and the main sources and types of evidence available (36). Unlike systematic reviews where assessments are made about the quality of the studies included and ‘apples are compared to apples’, scoping reviews are exploratory and utilize broader inclusion criteria such as including both qualitative and quantitative studies. Data are extracted and entered into a chart allowing the identification of common issues, themes, and gaps. The final step in a scoping review involves confirming the relevance of the findings through consultations with a group of experts or other stakeholders (37).

The initial search yielded 3,484 records. The titles and abstracts for these were read and inclusion and exclusion criteria applied. The title and abstract screen eliminated 3,447 records leaving 37 articles for review. First reading of the remaining articles led to the exclusion of eight more publications (four dissertations; three focused on IPV perpetrators; one newsletter blurb introducing the development of a new training for IPV and co-occurring mental health and substance use problems) leaving 29 articles.

**Fig. 1:**
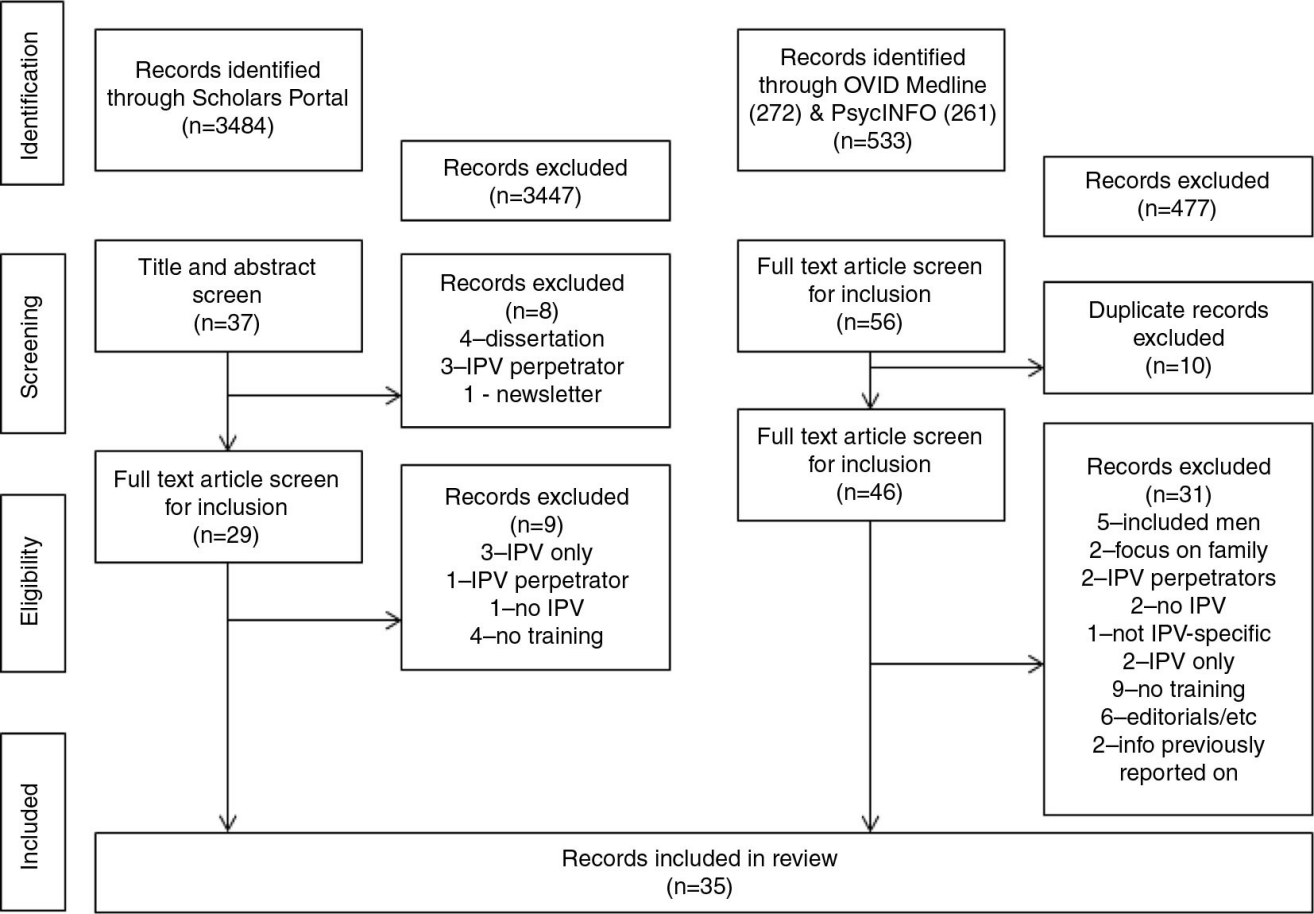
Records reviewed.

The remaining 29 articles were read in their entirety by the two authors. At this point nine additional articles were excluded; three because the focus was on IPV with no reference to either mental health or substance use, one because the focus was on IPV perpetrators, one because the focus was on substance use with no reference to IPV, and four because there was no reference to either treatment/education/training (Fig. 1).

The second search yielded 533 records (OVID Medline: 272 and OVID PsycINFO: 261). The titles and abstracts were read and the same inclusion and exclusion criteria applied, leading to the exclusion of 477 articles. Twenty-four articles from OVID Medline and 32 articles from OVID PsycINFO were left for review, however 10 of these were duplicates, leaving a total of 46 articles for final review.

The 46 articles were read in their entirety by both authors and an additional 31 articles were excluded; five combined male and female victims, two focused on family treatment, two combined victims and perpetrators, two because there was no IPV, one did not distinguish between other forms of trauma and IPV, two did not include co-occurring mental health and/or substance use problems, nine lacked reference or content relevant to treatment/education/training, six were editorials or commentaries, and two were retrieved in our earlier search (Fig. 1).

This review is based on the final sample of 35 articles (2005–2014) each of which referenced IPV and mental health and/or substance use plus treatment/education/training. Data extracted included: sample size and description, key findings, and recommendations (Tables 1, 2 and 3).

**Table 1:** Articles reviewed – IPV, mental health (MH), and substance use (SU)

Author, publication year	Sample size	Sample description/methods	Key findings	Recommendations provided
Cocozza et al., 2005 (39)	2,006 women	SAMHSA: quasi-experimental design with data collected from a convenience sample of women with co-occurring disorders and histories of sexual and/or physical abuse that entered integrated trauma informed treatment programs and comparative service-as-usual programs across the USA	Treatment improved outcomes in drug use and PTSD symptoms Integrated counseling significantly related to positive outcomes	×
Cohen et al., 2013 (40)	288 women	Secondary analysis of a randomized intervention trial comparing Seeking Safety (SS) and Women's Health Education (WHE) with women recruited from 7 community SU treatment centers across the USA	SS was not superior at reducing IPV risk at 1-year follow-up Women abstinent at baseline in SS significantly less likely to report IPV at follow-up SS is likely most effective for reducing future IPV in participants who are abstinent	✓
Domino et al., 2005 (41)	2,006 women	See SAMHSA study above	Intervention is more cost-effective than treatment as usual	×
Edwardsen et al., 2011 (42)	73 health care providers (HCP)	Pre/post evaluations of improved knowledge, attitude and efficacy of Veteran HCPs from 4 sites in New York State	Participants considered IPV a serious issue but felt they did not adequately address it Increase in HCPs knowledge and efficacy but no changes in attitude Preferred mode of learning: interactive activities, then lecture, then group work, then films	✓
Herz et al., 2005 (38)	415 health care providers	All IPV/sexual assault program staff, psychologists and psychiatrists, random sample of MH practitioners, all alcohol and drug abuse counselors in Nebraska, USA	IPV advocates had the most training and felt the most prepared; SU counselors had the least training and felt the least prepared Respondents felt their training was adequate and there was little need for more training	✓
Larson et al., 2005 (43)	2,729 women	See SAMHSA study above	High rates of physical health burden Providers should consider how this may hinder treatment participation	✓
Markhoff et al., 2005 (33)	9 sites	See SAMHSA study above	Recommendations and best practice guidelines for implementing trauma-informed care in MH and SU services	✓
Markhoff et al., 2005 (34)	1 site	See SAMHSA study above	A highly collaborative, inclusive, and facilitated change process can: effect services-integration within agencies strengthen integration within a regional network of agencies foster state support for services integration	✓
McHugo et al., 2005 (44)	9 sites	See SAMHSA study above	High exposure to stressful life events for study participants Services are more effective when they are gender-specific and trauma-informed	×
McPherson et al., 2007 (45)	324 women	Longitudinal, community-based study of mothers with severe mental illness recruited from 12 community agencies and 3 psychiatric units in a large Michigan city, USA	Women with MH and SU problems are more likely to report experiencing IPV	×
Morrissey et al., 2005 (46)	2,006 women	See SAMHSA study above	Intervention effects moderated by several person-level variables Women in high integrated sites had more positive outcomes (despite person-level characteristics) Integrated counseling appeared most effective in addressing SU and symptoms	×
Sabri et al., 2013 (49)	543 women	Cross-sectional study of African American (AA) and Caribbean (AC) women recruited from primary care, prenatal or family planning clinics in mainland USA and U.S. Virgin Islands	Severe physical and psychological abuse associated with high risk of intimate partner femicide (IPF) Positive association between comorbid PTSD and depression and IPF SU not significantly related to risk of IPF; PTSD was a significant mediator	✓
Savage and Russell, 2005 (47)	644 women	Subset of 2 SAMHSA sites (3 residential SU programs operated by a single multiservice agency in NY, USA and county-administered SU treatment program in California, USA) (see SAMHSA study above)	Support network characteristics moderate the effects of traumatic stress on trauma symptoms & MH (modest support) Women should be cautious relying on existing social support networks to help them heal	×
Savage et al., 2007 (48)	1,965 women	Subset of 6 SAMHSA sites (primarily SU treatment programs) (see SAMHSA study above)	Trauma severity significantly related to trauma distress but modest impact on MH and SU problems	✓

**Table 2:** Articles reviewed – IPV and MH

Author, publication year	Sample size	Sample description	Key findings	Recommendations provided
Crespo and Arinero, 2010 (50)	53 women	Randomized control trial with participants seeking IPV services from several organizations in Madrid, Spain	Both conditions reported significant improvements in PTSD symptomatology, depression and anxiety	✓
Hegarty et al., 2013 (51)	324 women (272) and doctors (52)	Cluster randomized control trial with family doctors and their patients from clinics in Victoria, Australia	No improvement on women's quality of life, safety planning and behavior or global mental health No differences in anxiety or comfort to discuss fear Depression symptoms significantly decreased in intervention Women in intervention asked more often about safety and children	✓
Howard et al., 2010 (52)	Unclear number of articles	Literature review on MH responses and interventions for psychiatric patients experiencing IPV		✓ (included articles from our review)
Laing et al., 2012 (53)	27 health care providers	Semi-structured interviews with action research project's working group (IPV and MH practitioners from Sydney, Australia)	Increased collaboration, built trust, shared sense of purpose, mutual commitment, built personal relationships (more imp for IPV than MH workers), developed institutional empathy Believed collaboration resulted in better client outcomes Developed a service agreement btw IPV and MH agencies Created new IPV-MH outreach worker position in IPV agency	✓
Miller et al., 2014 (54)	111 women	Randomized control trial of women recruited from general community and VAW shelters in urban and rural Midwestern USA and Southern Ontario, Canada	IPV victimization rates decreased for all mothers but significantly more for those in the intervention	✓
Nicolaidis et al., 2013 (55)	59 women	Pilot study evaluating a community-based intervention with pre/post intervention design with African American participants recruited from IPV services and word of mouth in Portland, Oregon, USA	Significant improvements in views about depression as well as depression severity, self-efficacy and self-management Increase in self-esteem and decrease in stress Modest dose response relationship i.e. those who participated least had least positive outcomes Outcomes not due to use of formal services or drug therapies for depression	✓
Sabri et al., 2013 (56)	431 women	See Sabri et al., 2013 above	More severe IPV associated with greater likelihood of MH problems, specifically PTSD and depression AA women with severe physical and psychological IPV and high risk for IPF significantly more likely to have co-occurring PTSD and depression Women with severe IPV who need MH care may be most at risk for underserved MH needs	✓

**Table 3:** Articles reviewed – IPV and SU

Author, publication year	Sample size	Sample description	Key findings	Recommendations provided
Bennett and O'Brien, 2007 (57)	255 women	Non-random sample of women seeking services from 1 of 6 agencies	Coordinated/integrated services associated with: greater self-efficacy in coping with and feeling less vulnerable to the effects of IPV greater perceived vulnerability to IPV decreased SU	×
Bennett and O'Brien, 2010 (58)	128 women	See Bennett and O'Brien, 2007 above	Care pathway does not have a substantial effect on outcomes where services are integrated/coordinated Women entering through IPV agencies had less positive SU outcomes at follow-up than those entering through SU doors Women entering through SU door have more complications than women entering through IPV door Suggests screening at intake only for IPV & SU is inadequate to capture long-term effects of IPV on SU recovery	✓
Brackley et al., 2010 (59)	n/a	Narrative review	Reviews TIPS 25 recommendations	✓
Fowler, 2007 (60)	102 women	New intakes and current VAW shelter residents	Incidence of SU 5–18% higher than noted in the file Supports the need for shelter-based SU assessment & intervention	×
Galvani, 2006 (61)	13 health care providers	Key informants drawn from a sample of professionals developing IPV & SU practices in England	Safety is primary consideration Treat the whole woman (not *just* her substance user) Appears SU providers fail to recognize & address IPV leaving women & children at risk	✓
Gilbert et al., 2006 (62)	34 women	Randomized controlled trial of adult women enrolled in an outpatient Methadone Maintenance Treatment Program who reported recent IPV and illicit drug use	Intervention effective in: reducing IPV & SU among women on methadone positive secondary outcomes (mental health distress& HIV) Intervention is both needed and feasible	✓ (with modification)
Gutierres and Van Puymbroeck, 2006 (63)	22 articles	Literature review	Complex relationship between trauma and SU Childhood violence creates a vulnerability to SU Childhood abuse and SU are independent but related risk factors for future adult violence victimization IPV and sexual assault in adulthood contribute to increased SU which leads to increased victimization SU treatment should address trauma and be designed specifically for women	✓
Humphreys et al., 2005 (64)	48 health care providers	Literature review/semi-structured key informant interviews with professionals working in IPV or SU policy or practice	Silos exist for many reasons (i.e. single issue focus, concerns about causality, complex needs, lack of knowledge and training, and fragmentation at government levels) Working together is more effective than working alone	×
Lipsky and Caetano, 2008 (65)	3,050 women and men	Sample drawn from 2002 National Survey on Drug Use and Health (cross sectional survey conducted annually in the USA)	Individuals who experience IPV are more likely to access alcohol treatment services Highlights potential to identify IPV in SU settings and provide referral and intervention services	×
Macy and Goodbourn, 2012 (66)	15 articles	Systematic literature review +Google/Google Scholar search and backward search of all documents	Promote successful collaborations (coordination, integration, linkage) btw IPV & SU treatment services, providers & researchers Interagency collaboration requires provider, director, agency and policy level strategies Challenges to collaboration include insufficient training, differences in service and treatment philosophies, limited financial resources, fragmented policies	✓ (included 3 articles from our review)
Macy et al., 2013 (67)	15 women	Exploratory qualitative study with women from SU treatment agency in Southwestern USA	Address the ways that co-occurring IPV & SU manifest in women's lives Children motivate women to seek help but women are reluctant to disclose IPV or SU for fear of losing their children	✓
Martin et al., 2008 (68)	71 health care providers	Survey sent to all 84 IPV programs in North Carolina, USA	Many women utilizing shelters have SU problems however not all shelters/staff are properly equipped to deal with SU problems	✓
Panchanadeswaran et al., 2008 (69)	416 women	Face-to-face, structured interviews with randomly selected woman from 14 Methadone Maintenance Treatment Programs in NYC, USA	Lower levels of perceived social support were significantly associated with physical aggression, sexual assaults and injurious attacks Highest levels of perceived social support were from significant others and lowest levels from friends Significantly lower levels of perceived social support for drug-abusing women in the context of IPV	✓
Schumacher and Holt, 2012 (70)	Unclear number of articles	Literature review	SA is common in women accessing IPV shelters; IPV shelter policies may bar women with active SA Preliminary evidence suggests that addressing both problems through parallel or integrated treatment may benefit women who access IPV shelters	✓ (excluded recommendations from articles in our review)

## Results

Of the papers reviewed, 14 examined the relationships among IPV, mental health, and substance use (33, 34, 38–49; Table 1), 7 focused solely on IPV and mental health (50–56; Table 2), and 14 looked at IPV and substance use (57–70; Table 3). One paper suggested topics for the purposes of cross-training in order to improve the care of victims of IPV (38) and another outlined the principles of trauma-informed care with the suggestion that these become core elements of all practice (33). One paper described the development of a curriculum on IPV and evaluated its impact on the knowledge, attitudes, and efficacy of participants (48). Reference to the development of a curriculum is made in one other paper focused on describing a systems’ change initiative (34), however, little detail was provided regarding curriculum content. Although education and training frequently figured among the recommendations in the reviewed articles, specific content of the proposed education or training was largely lacking. See Table 4 for the list of recommendations extracted.

**Table 4:** Recommendations extracted

Recommendation	Article
Integrate/coordinate/link services; use service agreements	34, 38, 48, 52, 53, 56, 58, 59, 66, 67, 68, 70
Consider IPV, MH & SU in IPV, MH & SU practice settings	34, 38, 40, 49, 56, 58, 63, 66, 67, 68, 70
Tailor services to individual including gender specific and culturally appropriate services	38, 49, 51, 55, 56, 59, 66, 67
Provide trauma-informed services, including assessment and treatment	33, 38, 48, 49, 56, 58, 67
Understand complex relationships among IPV, MH & SU (provider and client)	34, 49, 50, 56, 66, 67
Empower the consumer; emphasize strengths, self-esteem, resiliency	34, 48, 49, 63
Employ social cognitive theories/empowerment theories/psychoeducational interventions/transtheoretical model of behavioral change/motivational interviewing/cognitive behavioral program	50, 54, 62, 70
Consider multiple issues (retention, completion, relapse, practice issues, outreach, crisis intervention, physical disabilities, health problems, etc.)	34, 40, 61
Create safe, confidential & non-judgmental environment; use community spaces	34, 68; 55
Consider safety issues including lethality	49, 59, 67
Provide practical aid	50, 55, 69
Provide advocacy services	54, 55, 67
Provide peer-led services	34, 55
Standardize staff training; provide widespread training	38; 59
Strengthen women's social support networks	54, 69
Develop trust with women	55, 59
Address feelings of powerlessness, helplessness & guilt	63, 67
Use interactive and didactic curriculum elements	42
Develop shared sense of purpose, build relationships that promote trust, inclusive leadership, developing institutional empathy, specialist positions	53
Create a MH position in VAW agencies/VAW position in SU agency/etc.	53
Provide ongoing client-centered assessment and referral	58
Provide info about IPV/MH/SU in waiting rooms, etc.	59
Provide access/engagement	61
Use sensitivity in asking about abuse	63
Agency & policy changes are required	66
Training should be a required part of credentialing and licensing	66
Be aware of negative, critical supports	69

### IPV, mental health, and substance use

Of the 14 papers that examined the connections among IPV, mental health, and substance use (33, 34, 38–49), nine described aspects of a five-year, 14-center study in the United States, funded by the Substance Abuse and Mental Health Services Administration (SAMHSA), called the Women Co-Occurring Disorders and Violence Study (33, 34, 39, 41, 43, 44, 46–48). The SAMHSA projects were developed to ‘provide access to mental health and substance abuse services’ to women with co-occurring disorders and a history of sexual and/or physical abuse. However, the specific services and their provision were individually determined by each site making comparisons difficult (43).

Of the original 14 sites enrolled in the study, nine conducted program evaluations. Findings suggest that participants in both the treatment-as-usual and intervention conditions improved from baseline to six months in posttraumatic symptom severity, mental health status, alcohol problem severity, and drug problem severity. Significant outcomes for the intervention sites included greater improvement in posttraumatic symptom severity and drug problem severity compared with women in the treatment-as-usual sites. Furthermore, when compared to those in usual care, women in the intervention sites also showed some improvements in mental health status, although this did not reach significant levels. As noted, there were differences across the nine sites and in some sites greater improvement was shown for women in the treatment-as-usual condition when compared with the intervention arm (39). As the cost of services in the intervention sites was not more expensive than usual care and modest clinical improvements were shown in most sites, Domino et al. (41) suggested that the intervention was more cost-effective than usual treatment. Morrissey et al. (46) noted that at six months post-treatment, treatment effects were most positive for those with the most years of substance use.

The key recommendation to emerge from the cluster of SAMHSA studies was the benefit of integrated, trauma-informed care for women who experience co-occurring problems. However, little concrete information was provided about the specific core knowledge or skills required to meet this recommendation.

McPherson et al. (45) reported on a longitudinal study of mothers with severe mental illness and noted the high correlation among IPV, mental health, and substance use. Hospitalizations, symptoms, and alcohol and drug use at baseline were positively and significantly associated with IPV at six months follow-up. The authors concluded that given the high rates of IPV victimization, assessment, and referrals should be part of treatment or intervention plans for this population.

Although Herz et al.'s (38) needs assessment focused on all three sectors, their primary interest was to assess victim advocates, mental health, and addiction counselors’ knowledge of IPV and sexual trauma. A survey mailed to victim advocates, mental health, and substance use service providers in Nebraska, USA was designed to collect information about past training and education on IPV and sexual trauma. The authors noted there is little training on these issues (less than 10 hours) for mental health and substance use service providers, with the latter receiving the fewest hours of training. Yet, across all three groups, the majority of participants felt they had received adequate training. Specific content to educate providers about IPV and sexual trauma was included in the discussion section (incidence, impact, and overlap of IPV with sexual trauma, and behavioral health problems; understanding the dynamics of sexual assault in childhood and adulthood; IPV and offender behaviors; screening for histories, symptoms, or behaviors; support and intervention techniques; and effective referral, collaboration, and case management practices) however, little reference was made to cross-training on mental health or substance use problems. The authors acknowledged that the low response rate (29%) may have affected the generalizability of their findings.

Edwardsen et al. (42) also focused on training and education. They developed and evaluated an evidence-based curriculum on IPV for mental health providers working in veteran medical centers. The authors referenced topics included in other curricula, although specific details about their curriculum were lacking. The sole reference to content was that it emphasized the ‘overlap’ between IPV, mental health, substance use, and lethality. The day-long training was delivered to 73 participants along with validated pre and post-test measures of knowledge, attitudes, and self-efficacy (completed by 51 participants). Participants agreed that IPV was a serious issue and indicated the need for additional training. Post-test results indicated significant gains in knowledge and self-efficacy although no changes in attitudes were reported. Despite increased knowledge and participants’ indication they would likely use what they had learned, perceived barriers to incorporating knowledge into clinical practice remained high. Participants stated a preference for interactive sessions and lectures over group activities or films.

Sabri et al. (49) examined the risk for lethality among 543 African American and African Caribbean women who had experienced IPV. Participants in the cross-sectional study completed a number of standardized measures including the Danger Assessment, Women's Experiences of Battering, Severity of Violence Against Women (VAW) Scale, as well as measures of PTSD, depression, and alcohol use. The independent effects of severity of victimization, PTSD, depression, and alcohol use were examined relative to high or low risk of intimate partner femicide. Lethality risk was positively associated with co-occurring PTSD and depression but women's alcohol use was not related to increased risk for femicide.

Cohen et al. (40) were also interested in future and potentially lethal violence and compared two behavioral interventions designed for women with co-occurring IPV, PTSD, and substance use. Seeking Safety, a trauma-informed, integrated PTSD-substance use intervention that emphasizes safety issues, was compared with Women's Health Education, a psychoeducational group. IPV exposure, PTSD symptoms, and substance use outcomes were collected at one week, three months, and six or 12 months for 288 participants. No significant differences were noted between those who experienced IPV during the follow-up period and those who did not. IPV-related outcomes were similar across the two interventions with women currently using substances or with a substance-using partner, those with more lifetime exposure to traumatic events, and those who experienced physical or sexual assault in the 30 days prior, more likely to report IPV at follow-up. The authors suggested that Seeking Safety may be better suited to women who have achieved a period of abstinence while those with concurrent IPV and substance use may benefit from focused interventions that address trauma-related symptoms and substance use in a sustained way.

### IPV and mental health

Seven papers, all retrieved during the updated search, examined the relationship between IPV and mental health (50–56). Three (50, 51, 54) described Randomized Control Trials (RCT) of specific counseling interventions. One provided an overview of IPV and mental health outcomes including a review of known interventions (52). One outlined successful implementation of a peer-based treatment model that used Motivational Interviewing (55). One paper drew upon four related studies to delineate factors that contribute to successful IPV and mental health service collaborations (53). One examined mental health service utilization among a population of African American women who had experienced IPV (56).

Two RCTs found no significant differences in IPV-related outcomes between the intervention and control groups. Hegarty et al.'s (51) cluster RCT involved physicians and their patients. Physicians in the intervention group were trained to offer and deliver brief counseling (one–six sessions) to patients who screened positive for IPV. Twelve-month follow-up outcome measures included quality of life, safety planning, and mental health (including depression and anxiety). No between-group differences were noted on any measures except that women in the intervention reported fewer depressive symptoms. In the second RCT, Miller et al. (54) randomly assigned mothers to the intervention, Mothers Empowerment Program (MEP), a manualized evidence-based one-hour program that met twice weekly for 10 weeks (*n*=58), or a wait-list condition (*n*=62). Exposure to IPV was assessed for all participants at five weeks and again at six to eight months. MEP combined therapeutic services with advocacy and focused on violence and its effects, training in support of good mental health including conflict resolution, assertive communication, stress management, and emotional regulation in addition to advocacy. Episodes of violence decreased for women in both the intervention and comparison groups although those in the MEP experienced greater reductions. No measures of mental health were provided.

Crespo and Arinero (50) reported significant improvements in IPV survivors’ mental health in an RCT that compared two cognitive-behavioral programs. Both programs included psychoeducational components and relapse prevention; one program provided exposure procedures. Little difference in outcomes was reported between the two programs. Although not an RCT, Nicolaidis and colleagues (55) reported significant improvements in mental health outcomes for a community-based program using peer advocates, cognitive-behavioral therapy, and Motivational Interviewing. The program was developed with significant community input to specifically address the needs of African American survivors of IPV with depression symptoms (*n*=59).

Risk of lethality and mental health status were the focus of a study by Sabri et al. (56). Black women from the USA and US Virgin Islands who experienced both IPV and mental health problems were recruited to determine whether type and severity of IPV, including risk of lethality, were associated with PTSD, depression, and the use of mental health related resources (*N*=431). More than half of the women who experienced both PTSD and depression ‘were at increased, severe, or extreme danger of lethality’. Women who experienced more severe types of IPV were more likely to have co-occurring mental health problems. Neither IPV type and severity nor lethality risk predicted use of mental health resources.

Researchers frequently concluded their papers on co-occurring IPV and mental health with recommendations for improved collaboration. Factors that contributed to successful collaborations were examined in a paper by Laing et al. (53) that combined findings from four separate but related studies. These factors were identified as: commitment to building trust and a shared sense of purpose, personal relationships, the development of institutional empathy, and involved leaders who work to create a sense of inclusion.

### IPV and substance use

Fourteen papers examined the relationship between IPV and substance use (57–70). Two were general literature reviews (59, 63), one reported the prevalence rates of these co-occurring problems from a cross-sectional study (65), three were qualitative studies (61, 64, 67), three reported on interventions (57, 58, 62), three reported on substance use by women in VAW shelters (60, 68, 70), one examined perceived levels and sources of social support among substance using abused women (69), and one reviewed the literature on collaborations across IPV and substance use services (66).

Gutierres and Van Puymbroeck (63) reviewed the literature on the relationships of both childhood and adult victimization to substance use. Although the search strategy was not provided, the articles reviewed are thematically grouped into relationships examining childhood victimization and substance use (eight papers), adult victimization and substance use (seven papers), and childhood and adulthood victimization and substance use (seven papers). The authors noted that the relationships among these experiences are complex and causality cannot be determined. Two possible explanations for the increased risk of substance use among women who have experienced abuse were provided. The first suggests that a history of sexual abuse increases vulnerability to substance use; the second is an indirect path whereby abuse can lead to low self-esteem, depression, anxiety, guilt, and other psychological states that potentially increase vulnerability to adult victimization and self-medication through substance use or misuse. Clinical implications were reviewed and included a recommendation for cross-training for treatment providers; few suggestions about the training content were provided.

Brackley et al. (59) described a consensus report issued by SAMHSA in 1997 [Treatment Improvement Protocols (TIPS), number 25: Substance Abuse Treatment and Domestic Violence] noting that few settings had actually integrated the TIPS into practice. To improve implementation of the protocol, the authors outlined the important, potential role of both advanced practice and generalist nurses in screening and providing services to both victims and perpetrators. The article concluded with an outline of the specific knowledge, skills, and attitudes required to effectively intervene when IPV and substance use co-occur.

Three qualitative studies were reviewed. Humphreys et al. (64) and Galvani (61) conducted interviews with addiction treatment service providers, whereas Macy et al. (67) interviewed abused women in addiction treatment settings. Humphreys et al.'s (64) key informant interviews focused on the bidirectional relationships of IPV and substance use and the problems created by service delivery ‘silos’. They found five key themes highlighting the challenges of collaborative care: cultural clashes, a single issue focus, resource-related problems, governmental fragmentation, and providers’ lack of knowledge and training. Galvani (61) also interviewed key informants who worked in substance treatment settings. The 13 participants were asked about their IPV treatment experiences including access to, participation in, and retention in treatment. Participants reported a clear relationship between the two problem areas and noted that IPV impacts a woman's ability to access and complete treatment as well as increasing the potential for relapse. A number of practice-related issues (e.g. lack of training, policies, and perpetrator services) were also reported. Macy et al. (67) interviewed women in a trauma-informed substance abuse treatment agency about their perspectives on the connections between IPV and substance use. A majority of the 15 participants began their substance use early in life, prior to meeting their abusive partner. The authors suggested that women's progression from substance use to addiction may be facilitated by abusive partners and that women with addictions may benefit from understanding how violence triggers their substance use.

Gilbert et al. (62) and Bennet and O'Brien (57, 58) each reported on findings from interventions designed to enhance the safety and well-being of women who experience IPV and substance use problems. Gilbert et al. (62) conducted a feasibility study of an integrated group program for women in a methadone maintenance program that focused on both relapse prevention and increasing safety in intimate relationships. Participants received 12 group sessions and results indicated high rates of participation, attendance, and retention. Reductions in the number of IPV events as well as some improvements in substance use outcomes were also reported. In two papers, Bennet and O'Brien (57, 58) reported on the results of a demonstration project to explore collaboration and integration of IPV and substance abuse treatment services across six agencies in Illinois, USA. Two sites provided integrated services whereas four provided either violence or substance-related specific services with enhanced collaboration across sectors. All participants were screened for the co-occurring problem. The specific ‘door’ entered, or path to care, did not substantially affect outcomes. Most of the 128 individuals who completed the program and follow-up interview reported increased IPV related self-efficacy as well as a significant reduction in substance using days. The authors concluded that with enough training and support, providers could attend to both women's IPV and substance use needs. They suggested trauma-informed services are best suited to meeting those needs but did not elaborate on the training required.

Panchanadeswaran and colleagues (69) focused on perceived levels of social support of women on methadone, some of whom had experienced childhood abuse or IPV. Lower levels of social support were reported by women who experienced childhood abuse, IPV, or who used illicit drugs. Alcohol use did not affect perceived social support. Smaller social networks and lower perceived levels of social support may affect a woman's ability to recover from IPV and substance use.

Fowler (60), Martin et al. (68), and Schumacher and Holt (70) explored substance use problems among those in VAW shelters and programs. Fowler (60) focused on the extent to which 102 shelter residents screened positive for substance use problems using standardized tools (CAGE-AID, Simple Screening Instrument for Alcohol and Other Drug Use, Addiction Severity Index) and found approximately 68% of participants scored from moderate to high risk for substance use. Martin and colleagues (68) surveyed 71 of 84 North Carolina, USA, IPV programs about the ways in which they respond to women with co-occurring substance use problems. The authors reported that despite the frequency of substance use problems among women in these programs, more than half of the services had no written policy to guide practice. Key challenges to providing service to clients with substance use problems were noted as: women's refusal to participate in substance use treatment or the lack of available treatment options, the safety risks posed by housing women who are active substance users, and the risk of relapse and return to the abusive relationship of newly sober women. Participants spoke of the need for ongoing collaboration with treatment settings, open communication with clients about their substance use, and implementing substance use policies within IPV programs. The authors concluded that although staff largely recognized the high rates of co-occurring IPV and substance use among the women, few of the programs had trained staff to respond. Schumacher and Holt (70) synthesized the literature on the prevalence of substance using women in the shelter system and reviewed different treatment approaches for addressing co-occurring IPV and substance use including sequential treatment, linked treatment, and integrated treatment interventions. Challenges related to each of these options were discussed.

Once again emphasizing the importance of collaboration, Macy and Goodbourn (66) reviewed the literature on successful collaborations across IPV and substance use services as well as the many critical challenges facing these services. Included in the review were studies that evaluated the development, implementation, challenges, and outcomes of training programs designed to enhance collaboration.

## Discussion

The relationships among IPV, mental health, and substance use are complex and helping the women who experience these co-occurring problems presents a challenge to health and social service providers. Despite the high prevalence of co-occurring IPV, mental health and/or substance use problems in women's lives, evidence-based practices for responding to these complex problems are still in their infancy. Although this review clearly demonstrates the growing recognition among providers and researchers of the extent of these co-occurring problems, concrete and substantial recommendations to improve practices in all three sectors are largely lacking.

Acknowledging the challenges women who experience co-occurring IPV, mental health, and/or substance use problems face in accessing appropriate timely care, the most frequently occurring recommendations focused on the need to develop better collaboration, coordination, and integration across IPV, mental health, and addiction treatment services (34, 38, 48, 52, 53, 56, 58, 59, 66–68, 70). Within these services, trauma-informed approaches were reported to be useful in helping frontline providers understand the complex relationships among these problems and were a suggested promising practice in the provision of client care (33, 38, 48, 49, 56, 58, 67). In addition, social and cognitive strategies to empower women, increase self-esteem, and help them develop strong social networks were reported to be helpful (34, 38, 49, 54, 63, 69). Within services developed, recommended are environments that emphasize safety issues and confidentiality, and providers who practice without judgment (34, 68) and have considered how they will approach client relapse and crises (34, 40, 61).

Missing, however, is a clear consensus on the knowledge and skills those working on the frontlines require in order to better help those women who experience co-occurring IPV, mental health, and/or substance use problems. Recommendations have acknowledged the need to address these co-occurring problems but, with the exception of Edwardsen et al.'s (42) topics, most were too vague for frontline providers to act on (e.g. use integrated, trauma informed practices; staff training is required; assess and refer for co-occurring problems). Although some shelters, mental health counseling, and addiction treatment settings have embedded screening protocols into their intake procedures, there was little evidence of what happens once a woman does disclose a co-occurring problem. The development of cross-sectoral collaborations and protocols, trauma-informed practices, clear policies and guidelines, and staff education and training were among the recommendations proffered, yet there are few published studies on the effectiveness of these strategies. Developing such studies will be challenging due to current models of health and social service delivery where women who experience co-occurring problems are required to parse out the complexities to identify a single priority issue in order to access services. Should IPV first be addressed, or depression and low self-esteem? Should dependence on pain killers or alcohol be the primary focus or the violence and abuse?

Results from the SAMHSA cluster of studies suggest that integrated services where problems are recognized as being inter-related hold some promise. Yet, in many jurisdictions, VAW shelters, mental health, and substance use treatment services are funded by different government ministries. Development of the trauma-informed, integrated services piloted in the USA would require changes or, at minimum, agreement across ministries, as well as changes to the various professional education/training programs.

## Conclusion

Women who experience co-occurring IPV, mental health, and/or substance use problems have complex needs; the relationships among these problems are intricate, multilayered, and bidirectional. It is clear that more education and training is required to help those working on the frontlines respond to those needs appropriately, effectively, and compassionately, yet the research evidence to guide practice is still nascent with few concrete recommendations we could extract to guide curriculum content development for frontline workers. To better address the learning needs of those who work with women who experience these co-occurring problems requires collaborative, cross-sectoral, and multidisciplinary cooperation and systematic evaluation of new education and training initiatives.

## Footnotes

1 Scholars Portal provides access to ‘millions of e-journal articles and an extensive collection of e-books, geospatial data, and social science data sets. The content you will find in Scholars Portal comprises the most complete multidisciplinary database of peer reviewed academic literature available anywhere …’ (www.scholarsportal.info accessed Feb. 15/13).

## Appendix 1

Literature Review Search Strategy

***Database: OVID Medline***

1. exp Battered Women/ (2295)
2. exp Domestic Violence/ (35831)
3. Spouse Abuse/ (6226)
4. IPV.ti,ab. (2894)
5. (abuse adj2 (spouse or spousal)).ti,ab. (304)
6. exp *Violence/ and (Women's Health/ or Women's Health Services/ or Women/ or Female/ or female*.mp. or women*.mp.) (28478)
7. (battered adj2 (women or female*)).ti,ab. (558)
8. (violence adj2 (interpersonal or domestic or partner or family or gender or “against women”)).ti,ab. (10271)
9. exp Mental Disorders/ (962594)
10. exp Substance-Related Disorders/ (229107)
11. exp Alcoholism/ (67407)
12. Behavior, Addictive/ (5682)
13. (alcoholism or addiction* or intoxication or habituation or dependence or “binge drinking” or ((substance or drug) adj2 (abuse or abuses or misuse))).ti. (83086)
14. exp *Narcotics/ (69148)
15. exp Anxiety Disorders/ (68410)
16. exp Depressive Disorder/ (82053)
17. depressive disorder*.ti,ab. (21618)
18. depression.ti. (69354)
19. exp suicide/ (48885)
20. exp curriculum/ (65127)
21. exp Education, Professional/ or exp Education, Continuing/ (241377)
22. exp Inservice Training/ or exp Staff Development/ (24297)
23. (training or trauma-informed or trauma informed).ti,ab. (250116)
24. “best practice”.ti,ab. (6485)
25. Health Knowledge, Attitudes, Practice/ (74023)
26. exp Delivery of Health Care, Integrated/ (8718)
27. Diagnosis, Dual/ (2922)
28. Benchmarking/ (10322)
29. (cooccur* or co-occur* or co-exist* or coexist* or concurren* or dual).ti,ab. (296933)
30. 1 or 2 or 3 or 4 or 5 or 6 or 7 or 8 (48145)
31. 9 or 10 or 11 or 12 or 13 or 14 or 15 or 16 or 17 or 18 or 19 (1138761)
32. 20 or 21 or 22 or 23 or 24 or 25 or 26 or 27 or 28 or 29 (851007)
33. 30 and 31 and 32 (1018)
34. limit 33 to (English language and year=“2010–Current”) (298)
35. remove duplicates from 34 (272)

***Database: OVIC PsycINFO***

---------------------------------------------------------------------------------------------------------------------------------------------------------

1. exp Battered Females/ (1277)
2. exp Domestic Violence/ (5516)
3. exp Partner Abuse/ (5800)
4. exp Intimate Partner Violence/ (3910)
5. IPV.ti,ab. (2376)
6. (violence adj2 (interpersonal or domestic or partner)).ti,ab. (9819)
7. (abuse adj2 (spouse or spousal)).ti,ab. (219)
8. exp *Violence/ and (female* or women*).mp. (9492)
9. (battered adj2 (women or female*)).ti,ab. (603)
10. exp Mental Disorders/ (248298)
11. exp Drug Abuse/ or exp Addiction/ or exp Drug Addiction/ (50321)
12. exp Mental Health Programs/ (3706)
13. (mental adj3 health).mp. (88604)
14. exp Alcoholism/ (8948)
15. (alcoholism or addiction* or intoxication or dependence or “substance abuse” or “drug abuse” or “substance use”).ti. (22910)
16. exp Narcotics/ (805)
17. exp Major Depression/ or exp “Depression (Emotion)”/ (62559)
18. exp Anxiety Disorders/ (36045)
19. depressive disorder*.mp. (14851)
20. depression.ti. (31895)
21. exp suicide/ (11361)
22. exp curriculum/ or exp curriculum development/ (45293)
23. exp personnel training/ or exp inservice training/ (5783)
24. (training or trauma-informed or trauma informed).ti,ab. (90983)
25. exp continuing education/ or exp professional development/ (10427)
26. exp best practices/ (2255)
27. exp health personnel attitudes/ (10568)
28. exp community mental health training/ (252)
29. exp Mental Health Services/ (19505)
30. exp Health Care Services/ (58131)
31. exp Job Knowledge/ (739)
32. exp Dual Diagnosis/ (938)
33. (cooccur* or co-occur* or co-exist* or coexist* or concurren* or dual).ti,ab. (43932)
34. best practice*.ti,ab. (7588)
35. 1 or 2 or 3 or 4 or 5 or 6 or 7 or 8 or 9 (16886)
36. 10 or 11 or 12 or 13 or 14 or 15 or 16 or 17 or 18 or 19 or 20 or 21 (364948)
37. 22 or 23 or 24 or 25 or 26 or 27 or 28 or 29 or 30 or 31 or 32 or 33 or 34 (242591)
38. 35 and 36 and 37 (731)
39. limit 38 to (English language and year=“2010–Current”) (261)
